# A Mixed Method Study of Indian Mothers Assessing Impact of Lockdown in the Understanding and Burden of ADHD in Their Child

**DOI:** 10.1192/bjo.2022.232

**Published:** 2022-06-20

**Authors:** Prajakta Patkar

**Affiliations:** Topiwala National Medical College and BYL Nair Charitable Hospital, Mumbai, India

## Abstract

**Aims:**

To study the impact of the lockdown (pandemic) in the mother's understanding of the child's disorder (ADHD) and the burden faced by her.

**Methods:**

A mixed method design with a combination of a qualitative and quantitative approach. An in depth in-person semi structured interview with the participant mother was conducted as the qualitative part and the quantitative part of the study consisted of burden assessment by the Zarit Caregiver burden scale pre and post pandemic. The responses were transcribed and themes were identified

**Results:**

As far as understanding of the disorder was concerned, the major themes identified were “Knew about the child's problems from teachers but online schooling made me see the child's issues in person” and “Knew about the illness but more time led to more bonding and more understanding”. When questioned about the burden faced, the major themes that evolved were “Increased burden as I felt exhausted taking care of child 24/7” and “Increased burden as I felt angry and irritated with my child, the school and family”. The Zarit caregiver questionnaire revealed a statistically significant difference in the burden before and after pandemic with more number of mothers falling in the mild to moderate & severe category of burden after the commencement of the pandemic.

**Conclusion:**

COVID-19 pandemic increased the caregiver burden for Indian mothers of children with ADHD. They understood a lot more about their child's disorders by spending more time and devised different ways and means of helping their child in academic and other areas.

